# MicroRNA-185 Targets SOCS3 to Inhibit Beta-Cell Dysfunction in Diabetes

**DOI:** 10.1371/journal.pone.0116067

**Published:** 2015-02-06

**Authors:** Lidao Bao, Xudong Fu, Mingwen Si, Yi Wang, Ruilian Ma, Xianhua Ren, Haijun Lv

**Affiliations:** 1 Department of Pharmacy, The Affiliated Hospital of Inner Mongolia Medical University, Hohhot, Inner Mongolia Autonomous Region, China; 2 Department of Endocrinology, Liaocheng People’s Hospital, Shandong, China; 3 Department of General Surgery, Liaocheng People’s Hospital, Shandong, China; 4 Research Department, The Affiliated Hospital of Inner Mongolia Medical University, Hohhot, Inner Mongolia Autonomous Region, China; National Center for Scientific Research Demokritos, GREECE

## Abstract

Diabetes is the most common and complex metabolic disorder, and one of the most important health threats now. MicroRNAs (miRNAs) are a group of small non-coding RNAs that have been suggested to play a vital role in a variety of physiological processes, including glucose homeostasis. In this study, we investigated the role of miR-185 in diabetes. MiR-185 was significantly downregulated in diabetic patients and mice, and the low level was correlated to blood glucose concentration. Overexpression of miR-185 enhanced insulin secretion of pancreatic β-cells, promoted cell proliferation and protected cells from apoptosis. Further experiments using in silico prediction, luciferase reporter assay and western blot assay demonstrated that miR-185 directly targeted SOCS3 by binding to its 3’-UTR. On the contrary to miR-185’s protective effects, SOCS3 significantly suppressed functions of β-cell and inactivated Stat3 pathway. When treating cells with miR-185 mimics in combination with SOCS3 overexpression plasmid, the inhibitory effects of SOCS3 were reversed. While combined treatment of miR-185 mimics and SOCS3 siRNA induced synergistically promotive effects compared to either miR-185 mimics or SOCS3 siRNA treatment alone. Moreover, we observed that miR-185 level was inversely correlated with SOCS3 expression in diabetes patients. In conclusion, this study revealed a functional and mechanistic link between miR-185 and SOCS3 in the pathogenesis of diabetes. MiR-185 plays an important role in the regulation of insulin secretion and β-cell growth in diabetes. Restoration of miR-185 expression may serve a potentially promising and efficient therapeutic approach for diabetes.

## Introduction

Diabetes mellitus (DM) is a complex and multisystem disease characterized by elevated blood glucose levels resulting from either a lack of insulin production or resistance to insulin [1]. The rapidly emerging diabetes pandemic has been one of the most challenging threats to public health in the 21st century. It is reported that currently, 382 million adults worldwide are living with diabetes, and the estimate is projected to rise to more than 592 million by 2035 [2]. Life expectancy of a person diagnosed with type 2 diabetes at age 40 is estimated to be shortened by about 6–7 years, compared with people without type 2 diabetes [3]. To prevent and manage diabetes, at least 147 billion dollars were spent on diabetes health care in Europe in 2013, and North America and the Caribbean spent about 263 billion [4]. Thus, to completely understand mechanisms of diabetes development, and develop efficient therapy is of great importance.

MicroRNAs (miRNAs) are a family of small (∼22 nucleotide), non-coding single-strand RNAs which functions through negatively regulating a variety of gene expressions [5]. Mature miRNAs exert effects by integrating into an RNA-inducing silencing complex (RISC) and binding to specific complementary sites within 3’ untranslated regions (3’-UTR) of their target genes, to inhibit translation or directly induce degradation [6]. Growing evidences have implied that miRNAs are involved in pathogenesis of many diseases, including diabetes and its various complications [7], infections [8] and types of cancers [9]. Many studies have identified specific miRNA expression profiles of diabetes, and described the critical roles of miRNAs in pancreatic development and function [10]. For example, miR-375 is one of the most abundant miRNAs present in pancreatic islet cells and negatively regulates glucose-stimulated insulin secretion. Inhibition of miR-375 enhances insulin secretion, while miR-375 overexpression impairs the insulin secretory pathway by reducing expression of myotrophin [11]. MiR-375 also targets insulin gene expression and down-regulates phosphoinositide-dependent protein kinase-1, resulting in decreased insulin-induced phosphorylation of AKT and GSK3 [12]. Oppositely, miR-9 and miR-96 have inhibitory roles in insulin secretion. By targeting Onecut2 and Noc2 respectively, miR-9 and miR-96 up-regulate granuphilin, and negatively regulate insulin exocytosis [13, 14]. Although many miRNAs have already been identified, their roles in the regulation of key genes and signaling pathways associated with diabetes pathology still remain largely unknown.

MiR-185 and its role in cell biology first came to light when a connection was discovered between miR-185 expression and cancer progression [15]. Moreover, it was reported recently that miR-185-mediated inhibition of LDL uptake and HMG-CoA reductase activity is involved in dysregulation of cholesterol homeostasis, which is associated with various metabolic diseases, including diabetes [16]. However, the exact clinical-pathologic correlations and biological functions of miR-185 in diabetes have not been characterized. In this study, we confirmed the regulatory relationship between miR-185 and SOCS3, a member of the Stat-induced Stat Inhibitor. Our results showed that miR-185 could induce insulin secretion and suppress pancreatic cells apoptosis by directly targeting SOCS3. Restoration of miR-185 expression may have an important implication for the clinical management of diabetes.

## Materials and Methods

### Study subjects and definition of prediabetes and diabetes

Prediabetes, including impaired fasting glucose (IFG) and impaired glucose tolerance (IGT) was defined according to the ADA 2010 criteria [17]. IFG was defined as fasting plasma glucose (FPG) level of 6.1–7.0 mmol/L and a 2-h postprandial glucose (2-h PG) <7.8 mmol/L. IGT was defined as 2-h PG 7.8–11.1 mmol/L and FPG <7.0 mmol/L. DM was diagnosed when the FPG was ≥7.0 mmol/L, or the 2-h PG ≥11.1 mmol/L or HbA1c ≥ 6.5%.

A total of 34 type 2 diabetes patients who regularly visited affiliated hospital of Inner Mongolia Medical University between 2011 and 2013 were recruited in the study. Another 27 patients with IGT or IFG, and 30 normal subjects with normal glucose tolerance (NGT) were also recruited. Patients with the following conditions were excluded: (1) age ≥ 80 years; (2) malignancy; (3) serious liver disease (aspartate aminotransferase and/or alanine aminotransferase level >100 IU/L) or serious kidney disease (serum creatinine level >2.0 mg/dL); (4) acute heart failure. The study was carried out according to the institutional ethical guidelines and the use of human samples had been approved by the Medical Ethics Committee of Inner Mongolia People’s Hospital (IMP study ID G13–609025). The study was conducted according to the principles expressed in the Declaration of Helsinki. All samples were collected and analyzed with prior written, informed consent of the patients.

### Streptozotocin-induced mice diabetes model

This mouse study conformed to the Guides for the Care and Use of Laboratory Animals published by the U.S. National Institutes of Health and had been approved by the Medical Ethics Committee of Inner Mongolia People’s Hospital (IMP study ID A-Ms-14-3109113). A total of 8 male C57BL/6 mouse (6–7 weeks old, weighing 20–22 g) were purchased from Peking University Laboratory Animal Center (Beijing, China), housed in specific pathogen-free conditions and had free access to food and water prior to the experiment. After a week of acclimation, all animals were starved for 8 hours, and then 4 mice were randomly selected for a single intraperitoneal injection of streptozotocin (STZ, 180 mg/kg, dissolved in 0.1 M sodium citrate-hydrochloric acid buffer solution (pH 4.5)) to induce diabetes as previously described [18]. The remaining mice were injected with buffer solution without STZ as control group. Three days later, glucose was measured and mice with a blood glucose level of 16.7 mmol/L or above were diagnosed as diabetes. Islets were isolated 8 weeks later as previously described [19] and used further for RNA isolation and qPCR.

### Cell culture and treatment

MIN6 cells were purchased from AddexBio and cultured with DMEM medium containing 25 mM glucose, 15% fetal bovine serum and 5.5 mM 2-mercaptoethanol. Either miR-185-3p miRNA duplex mimics (miR-185 mimics), C. elegans-67 negative control duplex mimics (NC mimics), miR-185-3p miRNA hairpin inhibitor (miR-185 in) or C. elegans-67 negative control inhibitors (NC in) were purchased from Life Technologies, and transfected into cells by Lipofectamine 2000 according to the manufacturer’s instructions. pCMV SOCS-3 plasmid or pCMV vector was purchased from Addgene and transfected into cells by Lipofectamine 2000. The expression levels of miR-185 and SOCS3 were detected 48 h after transfection.

### Sample preparation and ELISA

Blood samples were directly collected in EDTA-anticoagulated tubes. Glucose was detected by routine laboratory method. Plasma SOCS3 was analyzed by ELISA according to the manufacturer’s instructions.

### RNA isolation and qPCR

Total RNA from the plasma samples was isolated using the miRNeasy mini kit with a modified protocol developed by Exiqon (RNA purification from blood plasma and serum). Total RNA from cells was isolated using Trizol reagent. For SOCS3 mRNA analyses, cDNA was generated from 1 μg total RNA per sample using the Verso cDNA synthesis kit and qPCR was performed using SYBR Green Master Mix. For mature miR-185 analyses, cDNA was generated using the Exiqon Universal cDNA synthesis kit and qPCR was performed using the TaqMan Universal Master Mix II. mRNA and miRNA expressions were normalized using detection of Actin and U44 respectively. Results are represented as fold inductions using the ^ΔΔ^Ct method.

### Western blot analysis

Cultured or transfected cells were lysed in RIPA lysis buffer (50 mM Tris/HCl, pH 8.0, 250 mM NaCl, 1% NP40, 0.5% [w/v] sodium deoxycholate, 0.1% sodium dodecylsulfate, 1% PMSF and 1×Phosphatase inhibitor cocktail). Cell protein lysates were centrifuged at 13000 rpm for 30 min at 4°C. 50 μg of cell lysate was subjected to 10% SDS/PAGE gel and transferred onto Immobilon PVDF membranes. Membranes were blotted with SOCS3, phospho-Stat3 (Tyr705), phospho-Stat1 (Tyr701) and phospho-Stat5 (Tyr694) antibodies purchased from Cell Signaling Technology at 4°C overnight, and subsequently incubated with HRP-conjugated secondary antibodies. Signals were visualized using ECL Substrates. GAPDH was used as a loading control.

### Glucose-stimulated insulin secretion (GSIS) assay

Cultured or transfected cells were seeded to a 96-well plate and incubated under basal glucose (3.3 mmol/L) and stimulatory glucose (16.7 mmol/L) conditions after starved overnight at 37°C. After 60 min, a sample was removed from each well for measurement of insulin by radioimmunoassay (RIA). Total insulin content was measured after sonication of cells in acid ethanol (2% H_2_SO_4_), followed by three freeze/thaw cycles and centrifugation for 5 min at 10000 × g. Insulin was measured in the supernatant by RIA and normalized to total DNA content extracted by a commercially available DNA purification kit.

### WST-1 proliferation assay

Cultured or transfected cells were seeded in a 48-well plate (4×10^4^ cells/well) in 200 μL growth medium supplemented with 20 μL WST-1 and incubated for 3 h at 37°C. The absorbance was measured with a spectrophotometer reader (wavelength 450 nm). Data shown correspond to mean values of three independent experiments measured in N = 6 per experiment.

### Cell viability assay

Cultured or transfected cells were seeded in a 96-well plate (2×10^3^ cells/well) in 100 μL growth medium. After 24 h incubation at 37°C, cell viability was determined by adding 50μL of CellTiter-Glo Luminescent reagent and by recording the luminescence for each well. Data shown correspond to mean values of three independent experiments measured in N = 6 per experiment.

### Apoptosis assay

Cultured or transfected cells were collected, washed with PBS and resuspended in binding buffer containing Annexin V-FITC and propidium iodide (PI). After 15 min of incubation at room temperature, samples were analyzed on a FACSalibur flow cytometer to determine rate of apoptosis.

### Luciferase reporter assay

Full-length of 3’-UTR of SOCS3 (1466 bp) containing the putative miR-185 binding site was amplified by PCR using the following primers:

wt-SOCS3 (forward): 5’- CCTCGGGAGTTCCTGGATCAGT-3’; wt-SOCS3 (reverse): 5’-ACGCGCTAATAGCTTGGAGCCT-3’.

The PCR product was then subcloned into a BamH I/Sal I site of the pGL3-basic luciferase reporter vector. A construct containing 3’-UTR of SOCS3 with a mutant seed sequence of miR-185 was synthesized using the following primers: mut-SOCS3 (forward): 5’- CCGGAGCAAAAGGGTCAGAGG-3’; mut-SOCS3 (reverse): 5’- ACCAGACGGCAGGAATTACCCA-3’.

All constructs were verified by DNA sequencing. HEK293 cells were plated in 96-well plates, and cotransfected with pGL3-constructs with or without miR-185 mimics. At 48 h after transfection, luciferase activity was detected using a dual-luciferase reporter assay system and normalized to Renilla activity.

### Statistical analysis

All values were expressed as mean ± SD and processed using the SPSS 13.0 software. At least three independent experiments were performed. The differences among the groups were estimated by Student’s t-test or one-way ANOVA. Spearman correlation analysis was used to determine the association between miR-185 level and blood glucose or SOCS3 level. Statistical significance was noted at P<0.05.

## Results

### MiR-185 is significantly downregulated in patients with diabetes and diabetic mouse model

**Fig 1:**
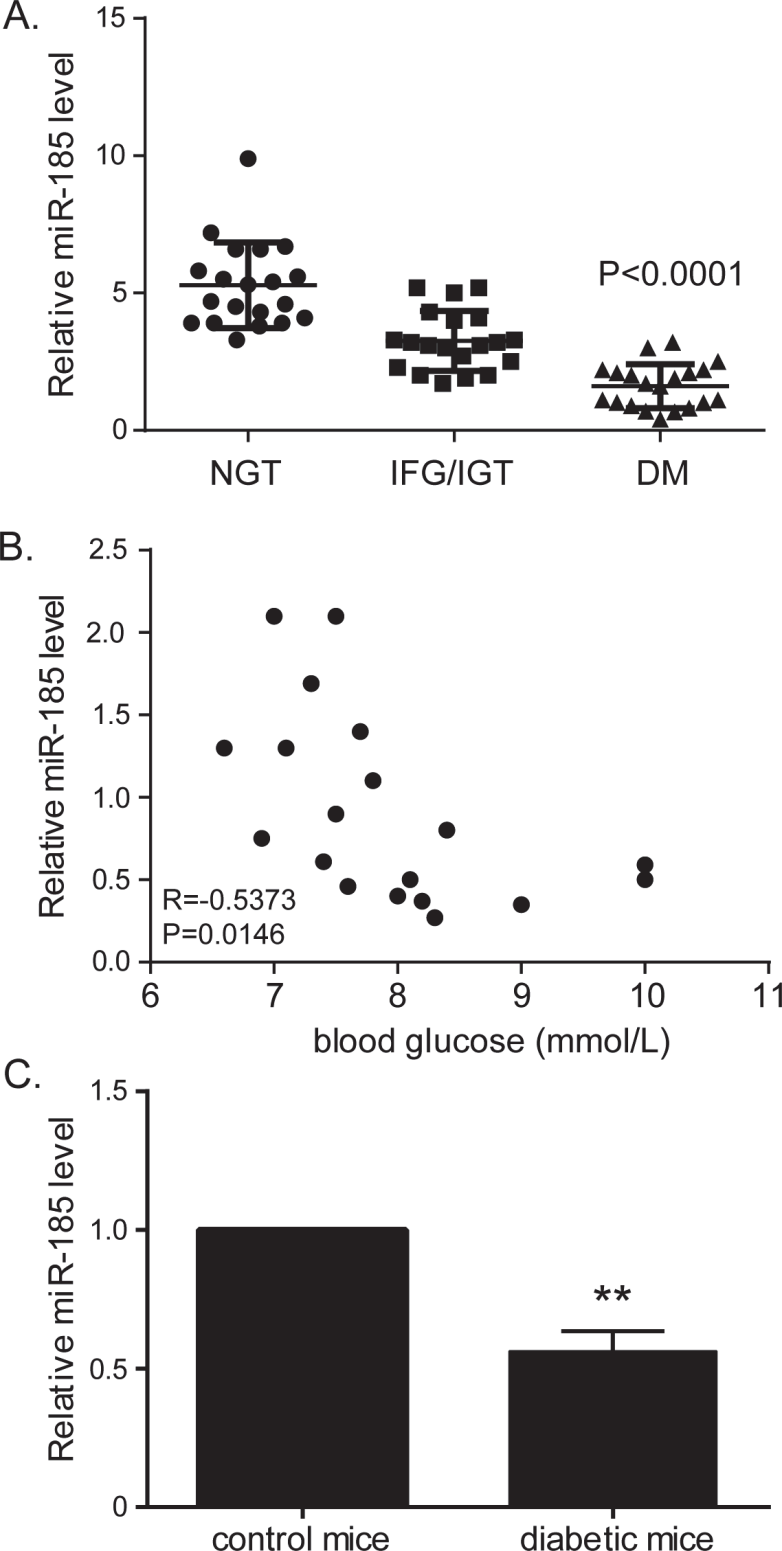
Expressions of miR-185 in plasma of patients with diabetes and isolated pancreatic islets of diabetic mice. MiR-185 expression was detected by qPCR and normalized by U44 expression. (A) Expressions of plasma miR-185 in patients with DM compared with those with IFG/IGT and NGT. (B) Correlation between miR-185 level and blood glucose determined by Spearman correlation analysis (R = -0.5373, P = 0.0146). (C) Expression of miR-185 is decreased in pancreatic islet isolated from diabetic mice compared with control mice. (**P<0.01; IFG: impaired fasting glucose; IGT: impaired glucose tolerance; DM: diabetes mellitus.

The expressions of plasma miR-185 in patients were determined by qPCR analysis. Diabetic patients had significantly lower level of miR-185, compared with those either with IFG/IGT or NGT (P<0.0001, Fig. 1A). Among patients with diabetes, a marked correlation between miR-185 level and blood glucose concentration was observed (R = -0.5373, P = 0.0146, Fig. 1B). Level of miR-185 was further determined in isolated pancreatic islets from 8-week-old diabetic mice induced by streptozotocin. Consistent with lower plasma level of miR-185 in diabetic patients, level of miR-185 was also significantly lower in pancreatic islets isolated from diabetic mice compared with control mice (P<0.0001, Fig. 1C).

### MiR-185 enhances insulin secretion and total insulin content

**Fig 2:**
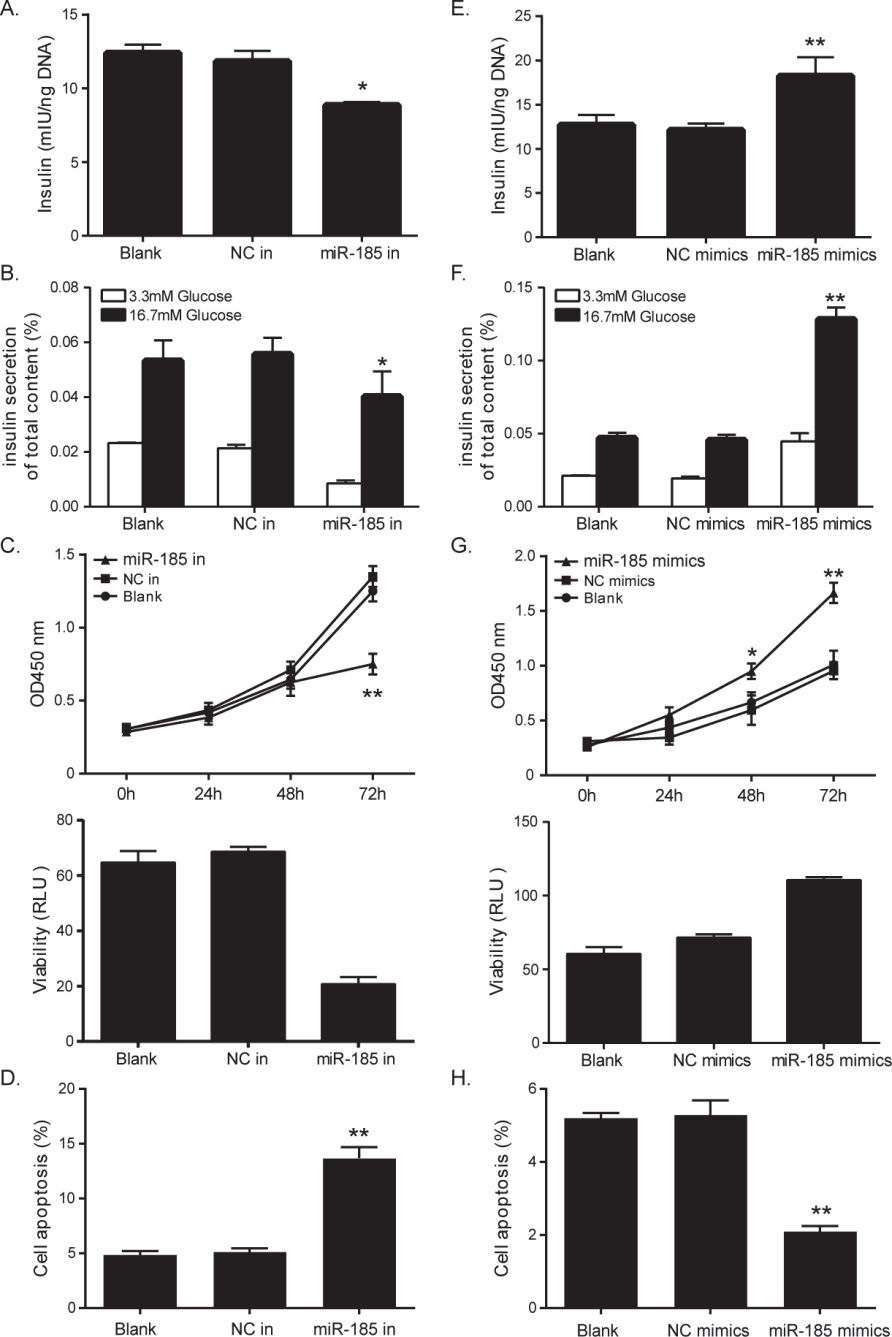
Effects of miR-185 on β-cell function and cell growth. Loss of miR-185 reduced total insulin content (A), inhibited glucose stimulated insulin secretion (B), reduced cell proliferation and viability (C), and stimulated cell apoptosis (D). Inversely, elevated miR-185 increased total insulin content (E), glucose stimulated insulin secretion (F), promoted cell proliferation and viability (G), and repressed cell apoptosis (H). (**P<0.01, *P<0.05, Figure is representative of 3 experiments with similar results.)

To analyze the functions of miR-185 in regulating metabolism, we induced alterations of cellular miR-185 level in the glucose-responsive murine pancreatic β-cell line MIN6 cells, and measured GSIS and insulin content. As shown in Fig. 2, insulin secretion in response to glucose stimulus was decreased in cells transfected with miR-185 inhibitor (Fig. 2A, B), and increased in cells transfected with miR-185 mimics (Fig. 2E, F) compared with the indicated controls. These data indicated that miR-185 was an inducer of glucose-stimulated insulin secretion.

### MiR-185 protects pancreatic cells from impairment

The ability of miR-185 on regulating cell growth was determined by proliferation assay, viability assay, and apoptosis assay. Loss of miR-185 significantly decreased the growth rate and viability of MIN6 cells compared with control cells (Fig. 2C). While cells treated with miR-185 mimics showed a dramatic increase in proliferation and viability (Fig. 2G). Apoptotic rate was analyzed by flow cytometry. The results showed that the rate of apoptosis was significantly higher in cells with lower level of miR-185 (Fig. 2D). However, it was remarkably decreased when miR-185 was overexpressed (Fig. 2H).

### MiR-185 directly targets SOCS3

**Fig 3:**
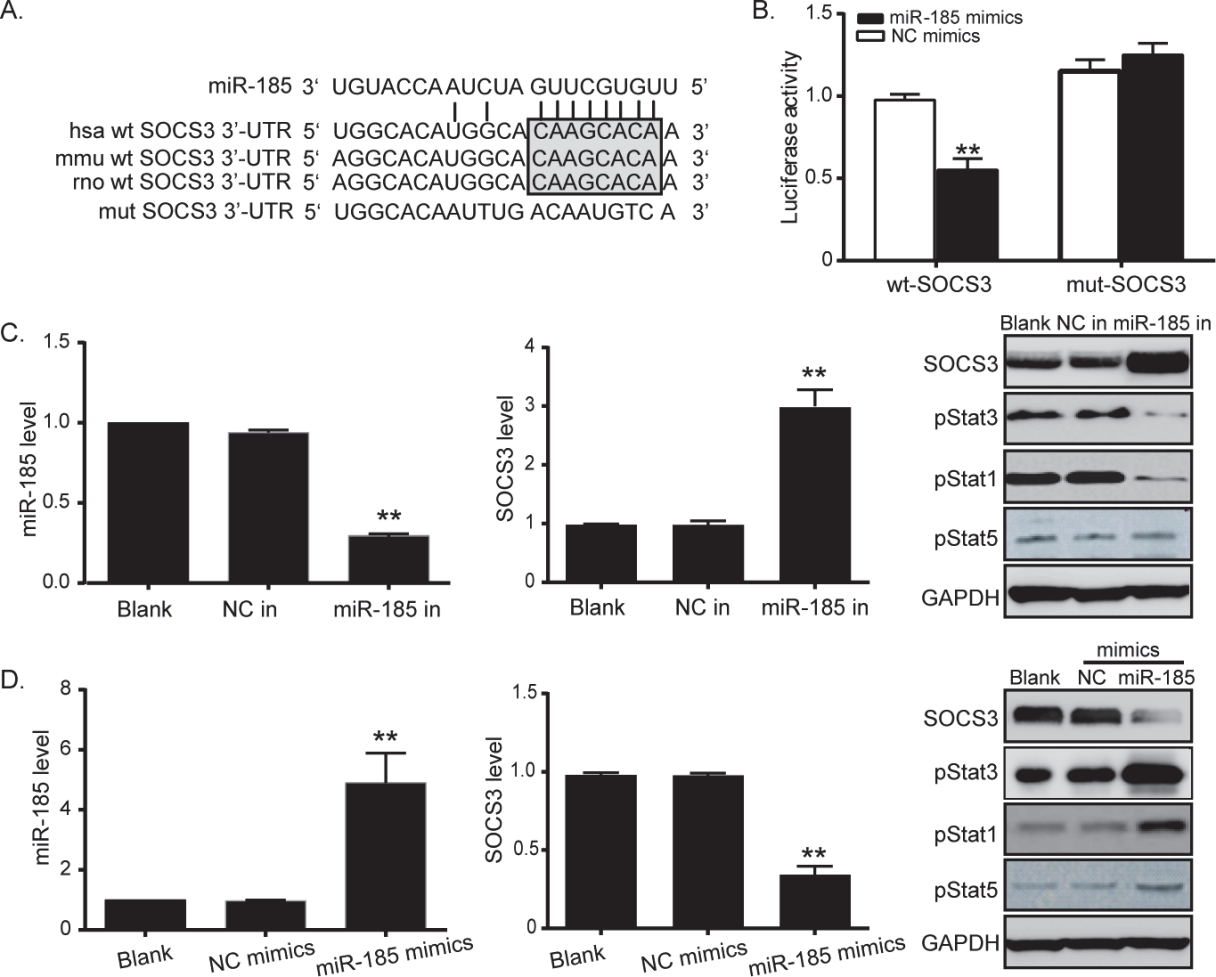
MiR-185 directly targets SOCS3 by binding to its 3’-UTR. (A) The predicted miR-185 binding site within SOCS3 3’-UTR in different species and its mutated version by site mutagenesis. (B) Luciferase reporter activities of wild type gene promoters were significantly repressed by elevated miR-185, whereas that of the mutant gene promoter was reversed to the normal level. (C) Left: Loss of miR-185 was induced by miR-185 inhibitor transfection. Middle: Reduction of miR-185 restored mRNA level of SOCS3. Right: Reduction of miR-185 restored protein level of SOCS3 and activated Stat3 pathway. (D) Left: Overexpression of miR-185 was induced by miR-185 mimics transfection. Middle: Elevated expression of miR-185 inhibited SOCS3 expression at mRNA level. Right: Elevated expression of miR-185 inhibited SOCS3 expression at protein level, and inactivated Stat3 pathway. (**P<0.01, *P<0.05, Figure is representative of 3 experiments with similar results.)

Four publicly available bioinformatic algorithms (TargetScan, Pictar, miRANDA, MICRORNA.ORG) were used to analyze target genes of miR-185. The results showed that SOCS3 was a theoretical target gene of miR-185 in different species, including human, mouse and rat (Fig. 3A). Further we subcloned the wild type SOCS3 3’-UTR fragment containing the miR-185 binding site or a corresponding mutant fragment into the pGL3-basic luciferase reporter vector respectively. Cotransfection with miR-185 mimics decreased luciferase activities of the wt-SOCS3 construct, but no change of luciferase activity was observed with the mutant construct (Fig. 3B). Consistently, PCR and western blot analyses showed that the mRNA and protein levels of SOCS3 were both dramatically up-regulated in miR-185 konckdown cells, but were downregulated when miR-185 was overexpressed (Fig. 3C and D). It is reported that SOCS3, a member of Stat inhibitor, is involved in negative regulation of the JAK/Stat pathway [20]. Thus we further detected activation of the Stat3 pathway. At the same time as SOCS3 was up-regulated by miR-185 inhibitor transfection, expression of phospho-Stat3 was significantly inhibited (Fig. 3C right). But when SOCS3 was repressed, phospho-Stat3 increased notably (Fig. 3D right). Other Stat family members, including Stat1 and Stat5 were also determined in response to the changes of miRNA-185. Levels of phospho-Stat1 followed the same trends as Stat3, but phospho-Stat5 remained unchanged in both the above two conditions. Taken together, these results suggested miR-185 could inhibit SOCS3 expression at transcriptional level.

### The effects of miR-185 on the pancreatic cells is mediated by SOCS3

**Fig 4:**
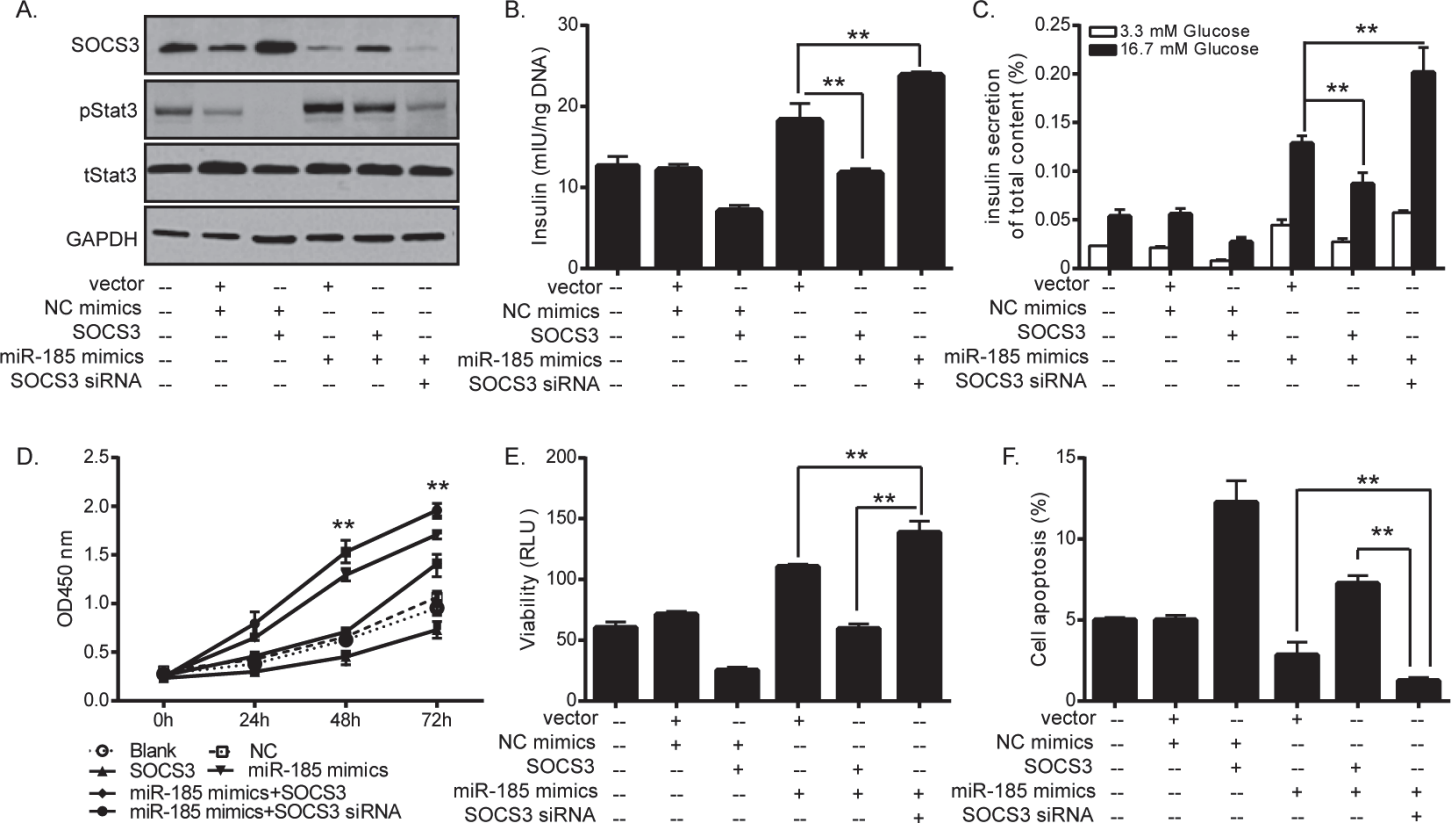
Functional effects of SOCS3 on pancreatic β-cells. (A) SOCS3 protein expression was effectively elevated by transfection with SOCS3 expression vector and knockdown by transfection with SOCS3 siRNA respectively. Stat3 pathway was suppressed by SOCS3. Elevated SOCS3 decreased total insulin content (B) and glucose-stimulated insulin secretion (C), inhibited cell growth (D), and induced cell apoptosis (E). Note that co-expression of miR-185 mimics and SOCS3 overexpression vector remarkably reversed the above inhibitory effects compared with transfection with SOCS3 alone and note the synergistic promotive effects induced by combination of miR-185 mimics and SOCS3 siRNA compared with either of them alone. (**P<0.01, *P<0.05, Figure is representative of 3 experiments with similar results.)

To further confirm the potential relationship between miR-185 and the downstream gene SOCS3, we next restored the expression of SOCS3 in miR-185-overexpressing MIN6 cells by cotransfection of SOCS3 expression vector and miR-185 mimics. When single SOCS3 gene was re-expressed (Fig. 4A), transfected MIN6 cells exhibited decreased glucose-stimulated insulin secretion (Fig. 4B and C), inhibited cell growth and viability (Fig. 4D and E), and induced apoptosis (Fig. 4F), which were in consistent with the inhibitory effects induced by downregulation of miR-185. Activation of Stat3 pathway was also suppressed by SOCS3. While co-expression of miR-185 and SOCS3 remarkably reversed the above inhibitory effects compared with transfection with SOCS3 alone.

Furthermore, SOCS3 expression was repressed by siRNA transfection (Fig. 4A) to block the SOCS3-miR-185 interaction. As shown in Fig. 4, when treating cells with SOCS3 siRNA in combination with miR-185 mimics, a synergistic inhibitory effect on SOCS3 expression was observed (Fig. 4A). Consistently, transfected cells shown increased insulin secretion (Fig. 4B and C), promoted cell growth and viability (Fig. 4D and E), and reduced apoptosis (Fig. 4F). Taken together, these results indicated that miR-185 played a protective role in pancreatic cells through regulating SOCS3 expression.

### The effects of miR-185 on the pancreatic cells involve Stat3 protein

**Fig 5:**
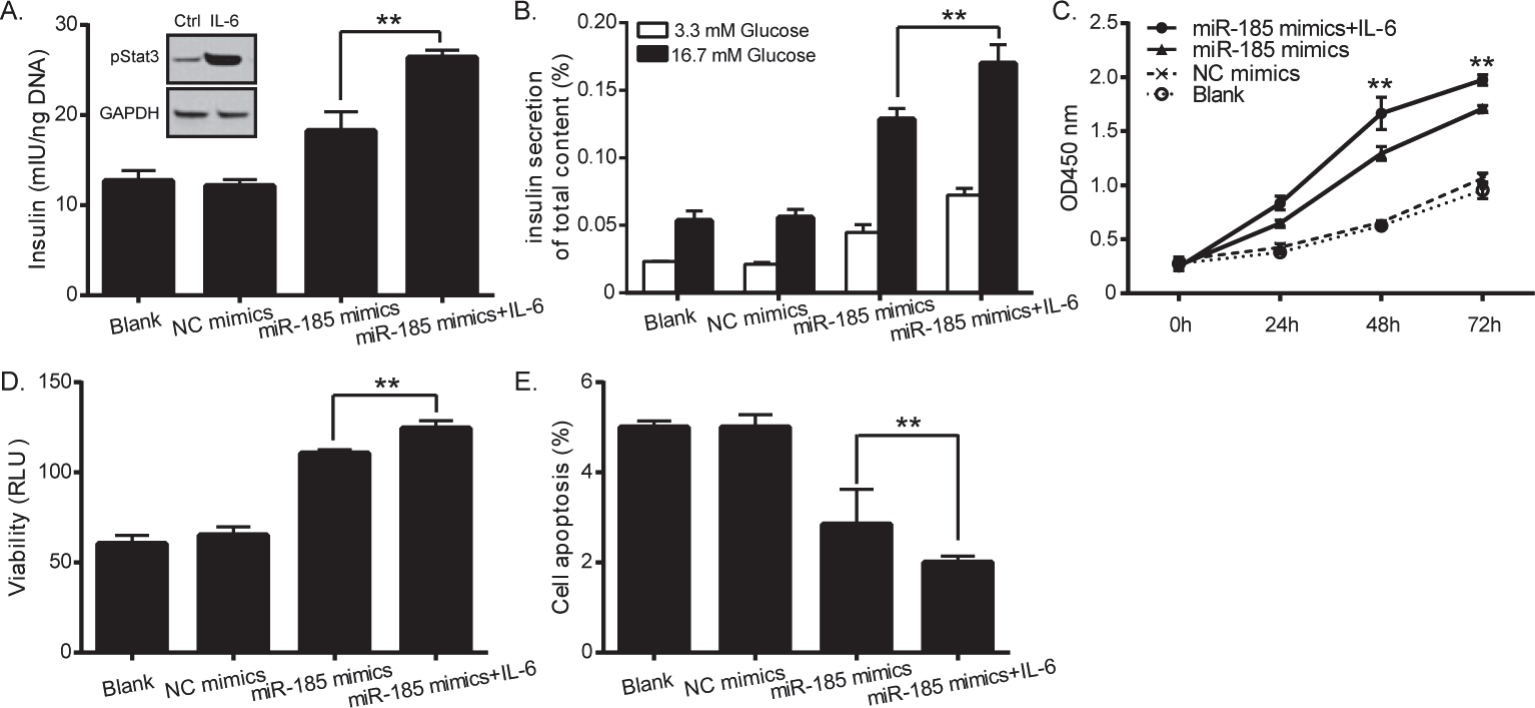
Functional effects of miR-185 on pancreatic β-cells involve Stat3 protein. Recombinant IL-6 was used to induce Stat3 activation (A top left corner). IL-6 treatment in miR-185 overexpressed cells resulted in more insulin secretion (B and C), cell growth and viability (D and E), and less apoptosis (F) compared with miR-185 overexpressed cells without IL-6 stimulation. (**P<0.01, Figure is representative of 3 experiments with similar results.)

As SOCS3 is a member of Stat inhibitor, thus we further investigated whether Stat3 protein participated in regulation of miR-185 on pancreatic cell function. Recombinant IL-6 was used to induce Stat3 activation (Fig. 5A top left corner), and treatment of miR-185 overexpressed cells with IL-6 resulted in much more insulin secretion (Fig. 5B and C), cell growth and viability (Fig. 5D and E), and even less apoptosis (Fig. 5F).

### Expression of miR-185 was clinically correlated with SOCS3

**Fig 6:**
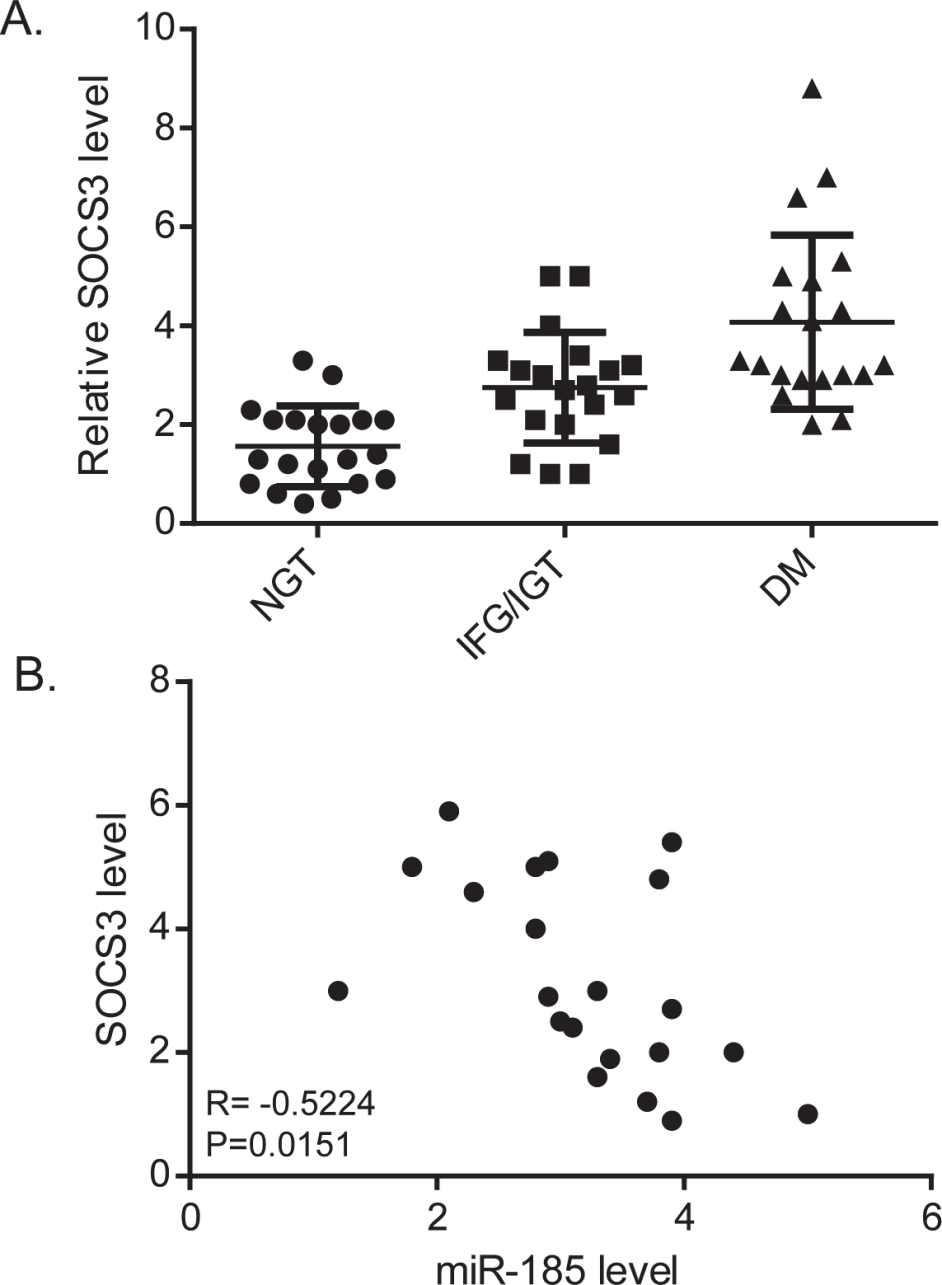
Expression of miR-185 was clinically correlated with SOCS3. (A) The plasma expression of SOCS3 was determined by ELISA. Expression of SOCS3 was higher in patients with diabetes than those with IFG/IGT or NGT. (B) Expression of miR-185 was inversely correlated with SOCS3 (Spearman’s correlation, R = -0.5224, P = 0.0151).

To confirm the clinical relevance between miR-185 and SOCS3 expression, we investigated the plasma expression of SOCS3 protein by ELISA in the recruited patients and controls. Expression of SOCS3 was much higher in patients with diabetes than those with IFG/IGT or NGT (Fig. 6A). Consistently, a significantly negative correlation between expression of miR-185 and SOCS3 was observed (R = -0.5224, P = 0.0151, Fig. 6B).

## Discussion

Diabetes is the most common and complex metabolic disorder. It is known that type 1 diabetes mellitus results from insulin deficiency, usually secondary to autoimmune β-cell destruction [21]; and type 2 diabetes mellitus requires defects in both β-cell function and insulin sensitivity [22]. But the mechanism driven β-cell dysfunction is largely unknown. Quite recently, using next-generation sequencing, a catalog of miRNAs was identified in human pancreatic islets and enriched β-cells, and the overlap between these miRNAs and type 2 diabetes pathogenesis was explored [23]. Studies on pancreatic development and function have implicated the critical role of miRNAs in these processes by the mechanism that miRNA could regulate a variety of genes pivotal for apoptosis and glucose-stimulated insulin secretion [10]. Another plasma-based microarray screening also described many circulating miRNAs associated with diabetes [24]. Of these deregulated miRNAs in diabetes specific miRNA profile, miR-185 was differentially expressed in diabetes. Further study showed that miR-185 regulated selective lipid uptake and is involved in major pathway of lipoprotein cholesterol metabolism [16, 25]. Based on these findings, we speculated that miR-185 might participate in diabetes development.

Our results obtained from qPCR validation in patients with different levels of glucose showed that there was a gradual decrease in plasma levels of miR-185 across categories of normal glucose tolerance, impaired fasting glucose/impaired glucose tolerance and manifest diabetes. Mouse model of diabetes induced by streptozotocin also presented significantly lower level of miR-185 in pancreatic islets. Further we restored miR-185 expression in the glucose-responsive murine pancreatic β-cell line, MIN6, and found that miR-185 inhibited β-cell dysfunction by enhancing insulin secretion and total insulin content, inducing cell growth, and inhibiting cell apoptosis. While when transfected with miR inhibitors to knockdown miR-185, MIN6 cells exhibited decreased insulin secretion and growth, as well as stimulated apoptosis. These findings suggested that miR-185 was involved in the processes of diabetes pathogenesis.

Previous study about miR-185 mainly focused on its regulation of cancer progression. In ovarian cancers, pediatric renal tumors and multiple breast cancer cell lines, miR-185 sensitizes resistant cancer cells to TRAIL-mediated apoptosis [26]. When miR-185 was up-regulated, the apoptotic gastric cancer cells were significantly increased in response to cisplatin or doxorubicin, demonstrating that miR-185 enhances the sensitivity of cells to chemotherapy [27]. MiR-185 also suppressed the proliferation potential and tumor growth in multiple types of cancer, including colorectal cancer [28], non-small-cell lung cancer [29] and ovarian cancer [26]. These investigations implied that miR-185 was possibly related to apoptosis. Our results, inconsistent with these conclusion derived from study based on cancer, show a promotive role of miR-185 in growth of pancreatic cells, indicating different molecular network functioning in diabetes compared with cancer.

The suppressor of cytokine signaling (SOCS) family was first discovered in 1997 and is referred to as signal transducer and activation of transcription (Stat)-induced Stat inhibitors [30]. It has been shown that SOCS3 is important in the development of leptin resistance, and inhibition of insulin is mediated by several SOCS proteins, especially SOCS3 [31, 32]. Elevation of intracellular SOCS3 levels blocks insulin signaling through ubiquitin-mediated degradation of IR substrate proteins, which share signaling cascades of JAK2/Stat3 with leptin receptors [32, 33]. In human aortic endothelial cells, increased cAMP induces SOCS3 protein expression, leading to leptin inhibition and IL-6-stumulated phosphorylation of Stat3 [34]. In mouse model of insulin resistance induced by high-fat diet, liver-specific knockdown of FGF-21 resulted in increased glycogenolysis and gluconeogenesis by activating glucose-6-phosphatase and phosphoenolpyruvate carboxykinase via the Stat3/SOCS3 pathway, which ultimately led to exacerbation of hepatic insulin resistance [35]. SOCS3 also plays important role in cellular proliferation and apoptosis. SOCS3 restoration by a demethylating agent in methylated pancreatic cells remarkably suppressed cell proliferation and induced apoptosis of cells [36]. In contrast, inhibition of SOCS3 promoted IFN-α-induced cell death and growth suppression in renal cell carcinoma [37]. Our results are consistent with these previous findings, and more importantly, we confirmed an important molecular relationship between miR-185 and SOCS3. Upregulation of miR-185 expression effectively suppressed SOCS3 expression at both mRNA and protein levels, whereas, downregulation of miR-185 induced SOCS3 expression, suggesting a potential inverse relevance between miR-185 and SOCS3 in diabetes. Consistently, a negative correlation between miR-185 and SOCS3 was observed in plasma samples of patients with diabetes. MiR-185 directly targeted SOCS3 gene through binding to specific complementary site within 3’-UTR of SOCS3 mRNA, and the effects of miR-185 on pancreatic cells is mediated by SOCS3. Taken together, these findings demonstrated that miR-185 played a protective role in diabetes, at least, in part due to directly inhibiting SOCS3 expression.

Although the insulin therapy has improved the lives of all people affected by diabetes, it is still one of the most challenging health problems worldwide, with high incidences of many complications. Our findings suggest that miR-185 significantly induces insulin secretion and pancreatic cell growth, by targeting SOCS3 and regulating Stat3 pathway. Patients with diabetes presented much lower level of miR-185, and the decreased level correlated with blood glucose concentration. Thus, our results implicate that miR-185 can be considered as a candidate for diabetes treatment, and the therapeutic effects of miR-185 warrants exploration.
